# Peptidase Regulation in *Trichophyton rubrum* Is Mediated by the Synergism Between Alternative Splicing and StuA-Dependent Transcriptional Mechanisms

**DOI:** 10.3389/fmicb.2022.930398

**Published:** 2022-06-17

**Authors:** Leonardo Martins-Santana, Monise Fazolin Petrucelli, Pablo R. Sanches, Nilce M. Martinez-Rossi, Antonio Rossi

**Affiliations:** Laboratory of Genetics and Molecular Biology of Fungi, Department of Genetics, Ribeirão Preto Medical School, University of São Paulo, Ribeirão Preto, Brazil

**Keywords:** *Trichophyton rubrum*, alternative splicing, StuA, peptidases, virulence

## Abstract

*Trichophyton rubrum* is the most common causative agent of dermatophytosis worldwide and uses keratinized substrates such as skin and nails as its main source of nutrition during infection. Its pathogenic character relies on colonization and viability maintenance at the target host sites. Since fungal physiology must adapt and respond to host conditions for the successful establishment of infection, biological mechanisms are constantly being triggered by *T. rubrum* to guarantee its survival in the host environment. The ability of this fungus to sense and modulate the secretion of specific proteases according to environmental pH signaling is considered as a pivotal virulence factor for effective invasion and persistence of infection in the host. Transcriptional regulation of genes encoding specific proteases, such as peptidases, is a key biological process that drives physiological modulation to meet fungal requirements. It accomplishes a robust balance among transcript isoforms that can be directed to perform distinct cellular functions. Thus, alternative splicing mechanisms are suitable for fungal cells to establish a balance toward reprogramming protein translation to impair or boost physiological conditions. In this study, we investigated the role of alternative splicing, especially intron retention events, in generating isoforms of virulence factors in *T. rubrum* mediated by transcriptional coordination of the protein StuA, a recently described transcription factor in this fungus. By analyzing the previous gene expression data provided by RNA-sequencing and after validation by reverse transcriptase quantitative polymerase chain reaction (RT-qPCR), we observed that two peptidase-coding genes (TERG_00734 and TERG_04614) could be direct targets of alternative splicing in the presence of keratin. Furthermore, protease isoforms generated by alternative splicing in *T. rubrum* were also detected in a co-culture with human keratinocytes, highlighting the role of these proteins in keratin deconstruction. Our results strongly suggest the influence of StuA on the regulation of virulence factors in *T. rubrum* and dermatophyte infections by triggering the transcription of the peptidase genes mentioned above in an alternative splicing-independent balance. The results elucidate how fungal cells drive alternate splicing to promote physiological adaptations and show that transcriptional regulation and virulence traits are robust elements required for dermatophyte infection.

## Introduction

Dermatophytosis is the most frequent type of mycosis and affects over 25% of people worldwide. They are commonly caused by a group of filamentous fungi known as dermatophytes. These species generally affect keratinized tissues, such as the skin, nails, and hair, by secreting enzymes to degrade these substrates into nutritional molecules, establishing a precise pH balance and providing cellular responses to maintain a suitable environment for their sustenance. Among the numerous species in this group, *Trichophyton rubrum* is the leading causative agent (Vermout et al., 2008; Leng et al., 2009; Martinez-Rossi et al., 2021).

The secretion of endo and exoproteases, such as subtilisins, fungalisins, aminopeptidases, dipeptidyl peptidases, and carboxypeptidases by dermatophytes is considered as a key factor for fungal invasion and its dissemination through the corneum stratum. To ensure establishment of infection, arthroconidia must adhere to the surface of the host tissue. This step is followed by germination and invasion of the keratinized tissue by fungal hyphae. During the invasion process, keratinized tissues are degraded into small peptides and amino acids that are captured by fungal transporters to be assimilated as nutritional sources. For the success of these steps, proteases play a pivotal role in achieving an orchestrated physiological balance as they are crucial for the obtainment of nitrogen species. Taking this into account, proteolytic enzyme activation can be studied as a virulence factor of dermatophytes (Monod et al., 2005; Leng et al., 2009; Baldo et al., 2012; Gräser et al., 2018).

Elucidating the mechanisms that are involved in fungal-host interactions, such as the coordinated processes comprising adhesion, penetration, and colonization of keratinized tissues, is gaining attention for developing new therapeutic strategies to treat dermatophytosis. In this scenario, we can consider transcription factors (TFs) as promising targets for antifungal therapy because of their involvement in diverse virulent aspects and fungal adaptive mechanisms to maintain cell viability (Bitencourt et al., 2021).

Recent studies published by our research group have highlighted the role of the APSES family TF StuA as a mediator of relevant mechanisms associated with the infectious process evoked by *T. rubrum*. Previous results have suggested that StuA may act as a potential therapeutic target for dermatophyte treatment. By constructing a StuA mutant strain of *T. rubrum*, our group showed that this protein is critical for morphogenesis, germination, adaptation, stress tolerance, virulence, and involvement in regulating the secretory mechanisms for keratin degradation (Lang et al., 2020). RNA sequencing of transcripts generated by the Δ*stuA* strain during growth on glucose or keratin also revealed that StuA could directly participate in the metabolism of carbon and oxygen reactive species, glycerol catabolism, cell wall construction, cell-to-cell adhesion, and immunomodulation, highlighting the central transcriptional role of StuA in regulating important cellular processes in *T. rubrum* (Bitencourt et al., 2021).

Transcriptional regulation is a critical factor that drives physiological processes in fungal cells. TFs play a tremendous role in different steps of signaling pathways and trigger mechanisms to activate or repress molecular responses depending on the biological conditions in which fungal cells are overwhelmed. In this sense, other aspects of molecular regulation are also relevant to the study of fungal response balance and are worthy of being studied. An example of such level is alternative splicing (AS). This ubiquitous mechanism occurs in pre-mature RNA transcripts to generate multiple isoforms of the same transcript, giving rise to different RNA/proteins depending on the cell requirements.

It is well established that intron retention (IR) events are among the most common events characterized by AS regulation in fungi (Muzafar et al., 2021). For this reason, AS-related studies have been continuously bringing to light how alternative RNA processing could elicit and modulate regulatory mechanisms relevant to fungal physiology, adaptation to fungal niches, pathogenicity, and drug resistance (Muzafar et al., 2021). Previous data showed that the mutant *T. rubrum* Δ*stuA* had a significant reduction in its keratinolytic activity (Lang et al., 2020). As a follow-up study, we have aimed to investigate the occurrence of AS-related isoforms of *T. rubrum* peptidase-coding genes and the role of StuA and AS in *T. rubrum* protease regulation in the presence of keratin.

The main goal of this study was to infer the role of proteases, particularly peptidases, in the initial steps of fungal colonization of host tissues in a process coordinated by transcription and AS regulation. By applying gene expression analysis (real-time quantitative polymerase chain reaction, RT-qPCR), we inferred that StuA is directly involved in the regulation of two protease-coding genes (TERG_00734 and TERG_04614). We also highlight the importance of AS as a second, StuA-independent regulation element involved in protease transcript processing in the co-culture of fungal cells and keratinocytes (HaCaT), suggesting that protease gene activation is pivotal for fungal invasion processes in contact with cells.

## Materials and Methods

### Analysis of Alternative Splicing

Alternative splicing (AS) was analyzed in R using the ASpli package (Mancini et al., 2021). Sequencing reads from the RNA-sequencing analysis were mapped to the *T. rubrum* reference genome using the STAR aligner (Dobin et al., 2013). The aligned reads were processed using the ASpli package to identify alternative splicing events (Mancini et al., 2021). Differential expression analysis was performed using the DESeq2 Bioconductor package (Love et al., 2014). The Benjamini–Hochberg adjusted value of *p* was set to 0.05, with a log2 fold change of ±1.5, to identify significantly modulated levels of transcript abundance (Martins et al., 2020; Bitencourt et al., 2021). Transcripts presenting an adjusted value of *p* lower than 0.05 were identified as differentially expressed. Only splicing events in genes that were not identified as differentially expressed or upregulated in keratin were filtered for analysis.

### Fungal Strains and Culture Condition

The *T. rubrum* CBS118892 strain (Centraalbureau voor Schimmelcultures, Fungal Biodiversity Center, Netherlands) was used as the wild type (WT). The null mutant strain (Δ*stuA*) was constructed as previously described (Lang et al., 2020). The WT and mutant strains were initially grown on malt extract agar solid medium (2% glucose, 2% malt extract, 0.1% peptone, 2% agar, pH 5.7) at 28°C for 20 days. Approximately 1 × 10^6^ conidia of each strain were inoculated into 100 ml of Sabouraud dextrose broth, and cultures were incubated at 28°C for 96 h with agitation (120 rpm).

The resulting mycelia were washed with sterile water and transferred to 100 ml of minimal medium (Cove, 1966) containing 70 mM sodium nitrate (Sigma-Aldrich, St. Louis, MO, United States) and 0.5% bovine keratin (m/v). Cultures were incubated for 24 and 48 h at 28°C with agitation (120 rpm). Biological material from three independent replicates was filtered and stored at −80°C until RNA extraction.

### Human Keratinocyte Strain and Culture Conditions

The immortalized human keratinocyte cell line HaCat was purchased from Cell Lines Service GmbH (Eppelheim, Germany) and cultured in RPMI medium (Sigma-Aldrich) supplemented with 10% fetal bovine serum at 37°C in a humidified atmosphere containing 5% CO_2_. Antibiotics (100 U/ml penicillin and 100 μg/ml streptomycin) were added to the culture medium to prevent bacterial contamination.

### Co-culture of Fungal Strains With Human Keratinocyte Assay

For the co-culture assay, 2 × 10^5^ HaCat cells/ml were plated in six-well plates and cultured in RPMI medium (Sigma Aldrich) supplemented with 5% fetal bovine serum at 37°C in a humidified atmosphere containing 5% CO_2_ for 24 h until cellular adherence to the plates was observed. Conidia suspensions (1 × 10^6^ conidia/ml) of *T. rubrum* (WT or Δ*stuA*) were added to the keratinocyte culture and incubated for 24 and 48 h. Uninfected cultured keratinocytes and *T. rubrum* conidia (wild type or Δ*stuA*) were used as control. The co-culture assay was performed in three independent replicates.

### RNA Extraction and cDNA Synthesis (RT-PCR)

Total RNA (from mycelia and co-culture) was extracted using an illustra RNAspin Mini Isolation Kit (GE Healthcare, Chicago, IL, United States). To disrupt the fungal cell wall in co-culture experiments, the samples were treated with a lysis solution containing 20 mg/ml of lysing enzymes from *Trichoderma harzianum* (Sigma-Aldrich) 0.7 M KCl, and 1 M MgSO_4_, pH 6.8, as described previously (Petrucelli et al., 2018). RNA concentration and quality were estimated using a NanoDrop ND-100 spectrophotometer (Thermo Fisher Scientific, Waltham, Massachusetts, United States).

Four hundred nanograms of total RNA was initially treated with DNAse I (Sigma-Aldrich) to avoid DNA contaminants in subsequent reactions. DNase I treatment was performed according to the manufacturer’s instructions. Subsequently, the samples were subjected to cDNA synthesis using the Platus Transcriber RNase H-cDNA First Strand kit (Sinapse Inc., Miami, FL, United States) according to the manufacturer’s instructions. The cDNAs obtained were analyzed for concentration and purity and then resuspended in dilutions of 70 ng/μl, which were used for quantitative PCR expression quantification.

### Real-Time Quantitative Polymerase Chain Reaction

**Figure 1:**
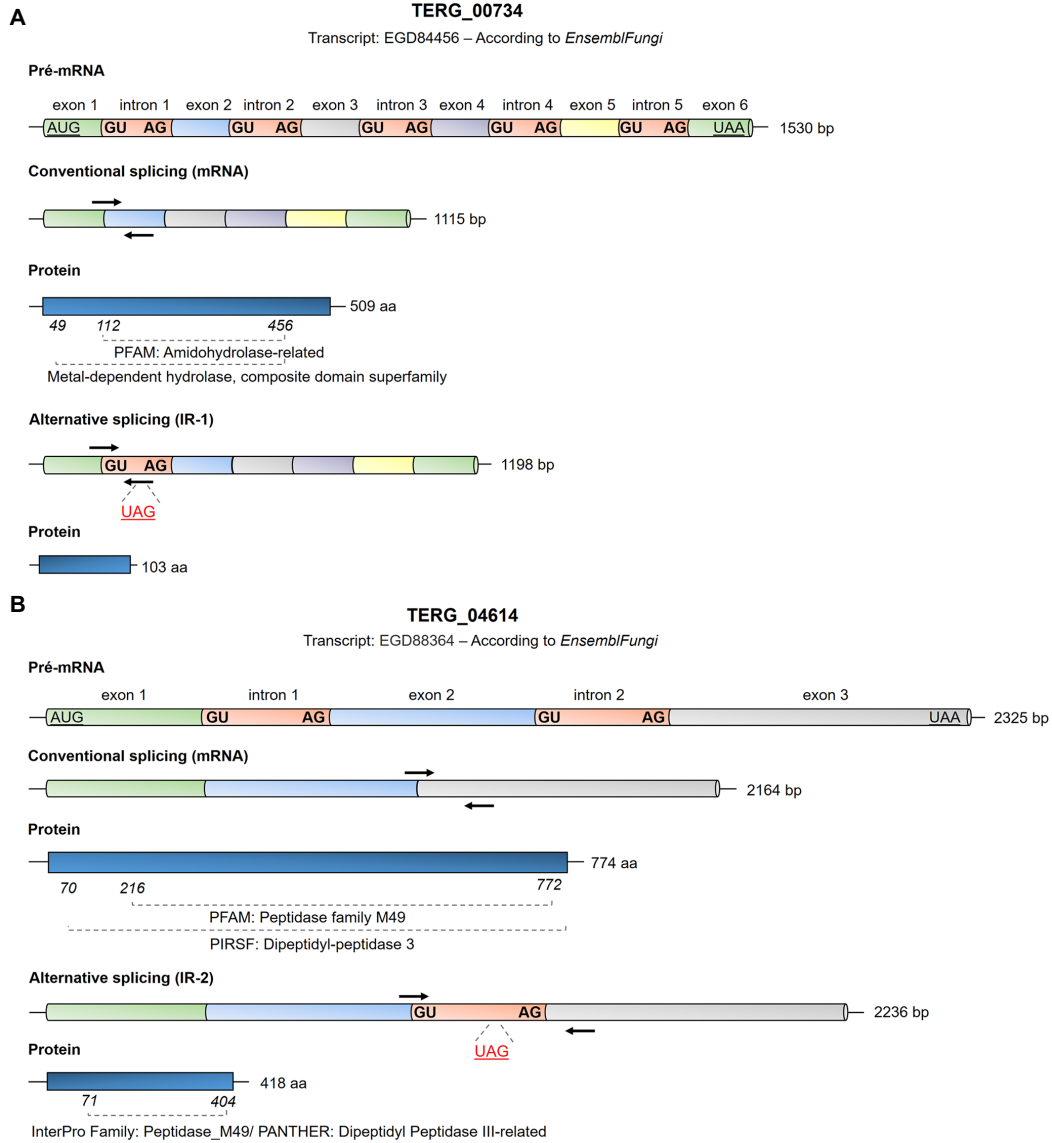
Schematic representation of the *Trichophyton rubrum* EGD84456 transcripts from TERG_00734 **(A)** and EGD88364 transcripts from TERG_04614 **(B)** based on the analysis using EnsemblFungi (https://fungi.ensembl.org/index.html). The conventional and alternative splicing events expected for these transcripts are shown as are the first and last nucleotides of each intron sequence. Initiation and termination codons are represented as underlying characters. Intron splice sites for each intron are highlighted in bold. Black arrows indicate primer annealing sites for transcript quantification by qPCR in this study in the mRNA panels. Details regarding the primers used in this study are provided in Supplementary Table S1. In the alternative splicing panel, the stop codons in the introns are shown as underlying red characters, indicating the blockage of translation for the specified transcript. The length of exons and introns are represented just for illustrative purposes, as it does not correspond to the actual size of the fragments.

Transcript quantification was performed using the QuantStudio 3 Real-Time PCR System (Applied Biosystems, Waltham, MA, United States) using the primers listed in Supplementary Table S1. The concentration of each primer was standardized for reaction efficiencies between 90% and 110%. A schematic representation of primer annealing for each protease gene is presented in Figure 1. Reactions were prepared using Power SYBR™ Green PCR Master Mix (Applied Biosystems) with ROX dye as a fluorescent normalizer according to the manufacturer’s instructions (Jacobson et al., 2018). For relative expression analysis, the 2^-ΔΔCt^ method was used, considering the gene *gapdh* of *T. rubrum* as the internal control for expression normalization (Livak and Schmittgen, 2001). The relative expression of the conventional splicing (CS) isoforms (without introns in the sequences) was normalized to the fungal strain conditions. Expression of IR events was quantified by adopting the quantification of the CS transcripts as a normalizer. For co-culture assays, expression analysis of the CS isoforms was carried out considering the fungal cells cultured in RPMI medium without HaCaT cells as a normalizer for co-culture quantification. The expression of the IR events was quantified using CS isoform expression as a normalizer for each strain condition. The results shown are the mean relative expression values from three independent replicates with standard deviations.

### Statistical Analysis of Gene Expression Quantification

As the results of transcript quantification were grouped into different AS event (CS and IR isoforms) presentations, statistical analyses were carried out using the unpaired t-test, and statistical significance was determined by the Holm-Sidak method considering *p* < 0.05, using. Significance is represented in the graphs by *p < 0.05, **p < 0.01, ***p < 0.001, and ****p < 0.0001. Statistical analysis and graphs were generated using GraphPad Prism software (version 6.0; GraphPad Software, San Diego, CA, United States).

### *In silico* Analysis of Protein Sequences for Peptidase Conserved Motifs Identification

The analyses of the isoform sequences, reading frames, conserved sites, and domains of the peptidases coded by TERG_04614 and TERG_00734 during AS events for CS and IR were carried out using *in silico* analysis tools. We used ExPAsy Translate Tool to identify the translated protein sequence of the analyzed transcripts.^1^ We searched for protein domains identification in virtual databases, such as Ensembl Fungi,^2^ Interpro (Hunter et al., 2009), and PANTHER (Mi et al., 2019, 2021). We drew a graphical representation of each isoform using the Illustrator for Biological Sequences software (IBS 1.0; Liu et al., 2015).

### Detection of IR Events in Agarose Gel

To detect IR events in the transcripts, end-point PCR (conventional PCR) was carried out using the primers listed in Supplementary Table S2. A total of 210 ng of cDNA from each strain and condition was used as a template for the reactions. Taq DNA polymerase from *Thermus aquaticus* (Sigma-Aldrich) was used to amplify the fragments according to the manufacturer’s instructions. Reactions were subjected to an initial denaturation at 94°C for 2 min, followed by 40 cycles of denaturation at 94°C for 1 min, at 55°C for primer annealing for 30 s, and at 72°C for 30 s for extension, and final extension at 72°C for 10 min. Amplicons were visualized on a 2% (w/v) agarose gel using SYBR® Safe DNA gel stain (Invitrogen, Thermo Fisher Scientific). Reactions were performed using primers for the CS and IR transcripts of each gene. IR primers were designed to flank intron regions, so amplification of fragments that anneal in coding regions was also expected during the reactions. The expected amplicon lengths are shown in Supplementary Table S2.

## Results

### AS Events Regulate Proteases Through IR Balance

By analyzing previous RNA sequencing data of *T. rubrum* Δ*stuA* (grown on keratin) published by our research group, we observed that some protease-coding genes are the targets of AS events through IR (Table 1). The *Trichophyton tonsurans* orthologous aminopeptidase TERG_06767 and dipeptidase TERG_00734 were upregulated in the RNA sequencing results, presenting differential IR transcript levels compared to the WT strain. IR events are addressed at introns 1 and 5, respectively, for these proteases in the identified transcripts. In addition, IR events also occur in the *Trichophyton verrucosum* ortholog dipeptidyl peptidase III TERG_04614 and *T. tonsurans*’ ortholog metallopeptidase TERG_05923, which present IR events both addressed at intron 2 of their transcripts.

**Table 1:** Quantification of Intron retention (IR) events in proteases found to be differentially and not differentially expressed in previous RNA sequencing.

Time point	ID	Gene product name	Log2FC DE	Log2FC IR
**Differentially expressed genes in RNA sequencing**				
24 h	TERG_06767 IR-1	Aminopeptidase (*T. tonsurans*)	2.62	1.96
48 h	TERG_06767 IR-1	Aminopeptidase (*T. tonsurans*)	2.52	1.54
48 h	TERG_00734 IR-1	Dipeptidase (*T. tonsurans*)	2.07	−0.94
**Not differentially expressed genes in RNA sequencing**				
24 h	TERG_04614 IR-2	Dipeptidyl peptidase III (*T. verrucosum*)		1.28
48 h	TERG_04614 IR-2	Dipeptidyl peptidase III (*T. verrucosum*)		1.04
48 h	TERG_05923 IR-2	Metallopeptidase (*T. tonsurans*)		2.38

ID, identification of the gene represented by each TERG; Log2FC DE, values of expression of differentially expressed genes mathematically represented as Log2 fold change comparing Δ*stuA* to the WT strain; and Log2FC IR, values of expression for intron retention events mathematically represented as Log2 fold change comparing Δ*stuA* to the WT strain.

In this study, we investigated the role of IR in two proteases identified *in silico*. We observed inconsistent transcription patterns for TERG_00734 and TERG_04614. We were interested in elucidating whether StuA and AS were involved in distinct regulatory mechanisms of *T. rubrum* in the presence of keratin.

For these purposes, we checked the presentation of both protease configurations at Ensemble Genomes.^3^ For TERG_00734, there was only one annotated transcript with five exons in the reverse strand. For TERG_04614, only one annotated transcript had three exons in the forward direction. In Table 1, an annotation of the IR events for TERG_00734 is shown at intron 5. However, because the orientation of the strand is in the reverse configuration, an IR event is believed to occur at intron 1 instead.

### AS Events by IR Result in mRNA With Premature Stop Codons

AS events induced by IR can result in mRNA with premature stop codons, consequently compromising protein translation. *In silico* analysis showed that the CS of TERG_00734 and TERG_04614 resulted in functional proteins with conserved domains (Figures 1A,B). When IR events occur, disruptions in the open reading frame result in mRNAs with premature stop codons. For TERG_00734, alternative processing in pre-mRNA with intron 1 retention generated a putative protein with only 103 amino acid residues (Figure 1A). For TERG_04614, retention of intron 2 during AS events produced a protein with 418 amino acid residues. Interestingly, even with intron 2 retention, part of the protein domain was maintained, raising the possibility of compromised functionality of this protein (Figure 1B).

### Peptidase Transcript Pattern in Keratin Is Dependent on StuA

**Figure 2:**
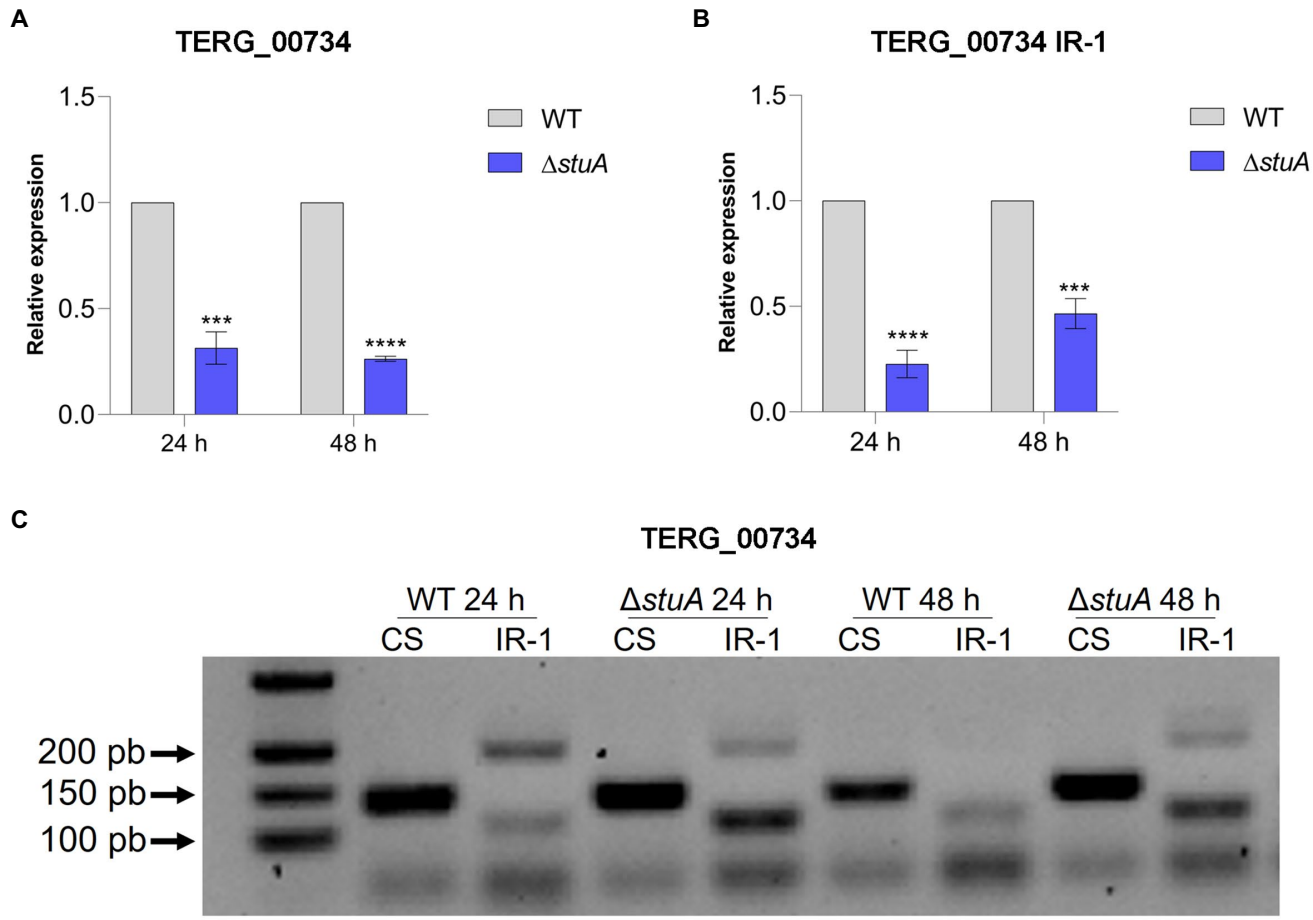
**(A)** Relative expression of the CS isoforms of TERG_00734 with WT strain transcript levels as the reference. **(B)** Relative quantification of the IR isoforms of TERG_00734 considering CS isoform transcript levels of each strain as references. **(C)** CS and IR (intron 1 retention—IR-1) isoforms in agarose gel after conventional amplification using cDNA of each strain as template. Statistical analyses for transcript quantification experiments were carried out using unpaired *t*-tests, and statistical significance was determined by the Holm-Sidak method considering ***p < 0.001 and ****p < 0.0001.

The peptidases evaluated in this study revealed a significant decrease in isoform transcripts submitted to CS after *stuA* gene disruption. As shown in Figure 2A, the absence of StuA resulted in lower levels of the TERG_00734 CS isoform after both 24 and 48 h of culture in keratin. In addition, it was observed that the absence of the StuA protein also resulted in a negative modulation of the transcript levels of the isoform, which were under IR events in keratin culture (Figure 2B). IR events for this transcript were detected in both strains after 24 and 48 h of culture, especially for the WT strain, and visualized as lanes of approximately 200 bp in an agarose gel (Figure 2C).

**Figure 3:**
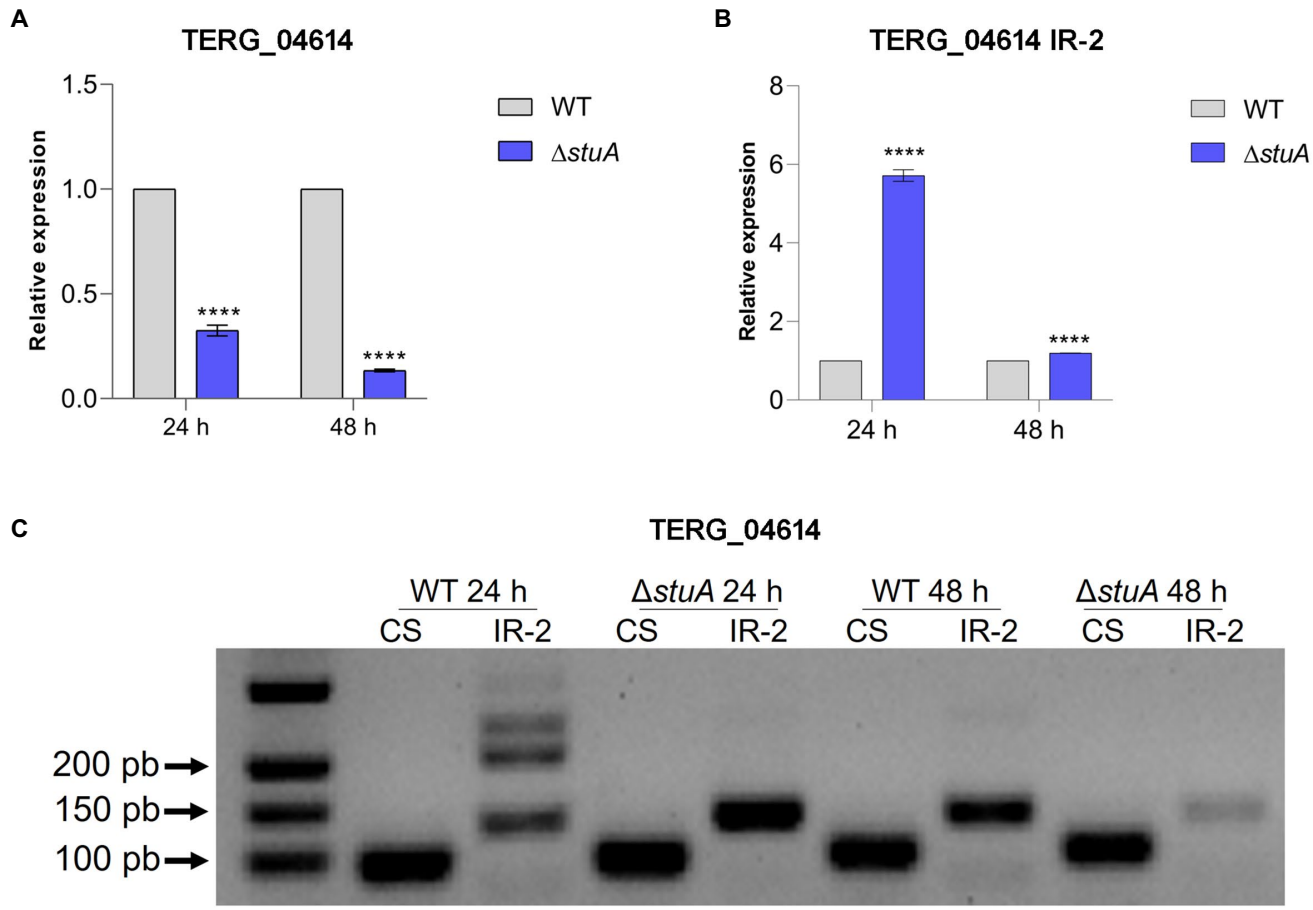
**(A)** Relative expression of the CS isoform of TERG_04614 with WT strain transcript levels as the reference. **(B)** Relative quantification of the IR isoforms of TERG_04614 considering CS isoform transcript levels of each strain as references. **(C)** CS and IR (intron 2 retention-IR-2) isoforms in agarose gel after conventional amplification using cDNA of each strain as template. Statistical analyses for transcript quantification experiments were carried out using unpaired *t*-tests, and statistical significance was determined by the Holm-Sidak method considering ****p < 0.0001.

Transcriptional analysis of the peptidase TERG_04614 followed the same pattern found previously, as disruption of the *stuA* gene promoted a decrease in the levels of the CS transcript isoform in keratin culture (Figure 3A). However, the absence of StuA seems to positively affect the abundance of the transcript under IR events after the AS process, as a relevant increase in the transcript levels of intron 2 of this gene can be detected mainly after 24 h of culture in keratin (Figure 3B). Interestingly, the observation of such IR events in agarose gel was only visualized after 24 h of keratin culture, in agreement with the increase in the detected levels of IR transcripts for this gene (a lane corresponding to approximately 200 bp; Figure 3C).

### Peptidase Transcription Is Boosted on Co-culture With HaCaT Cells

**Figure 4:**
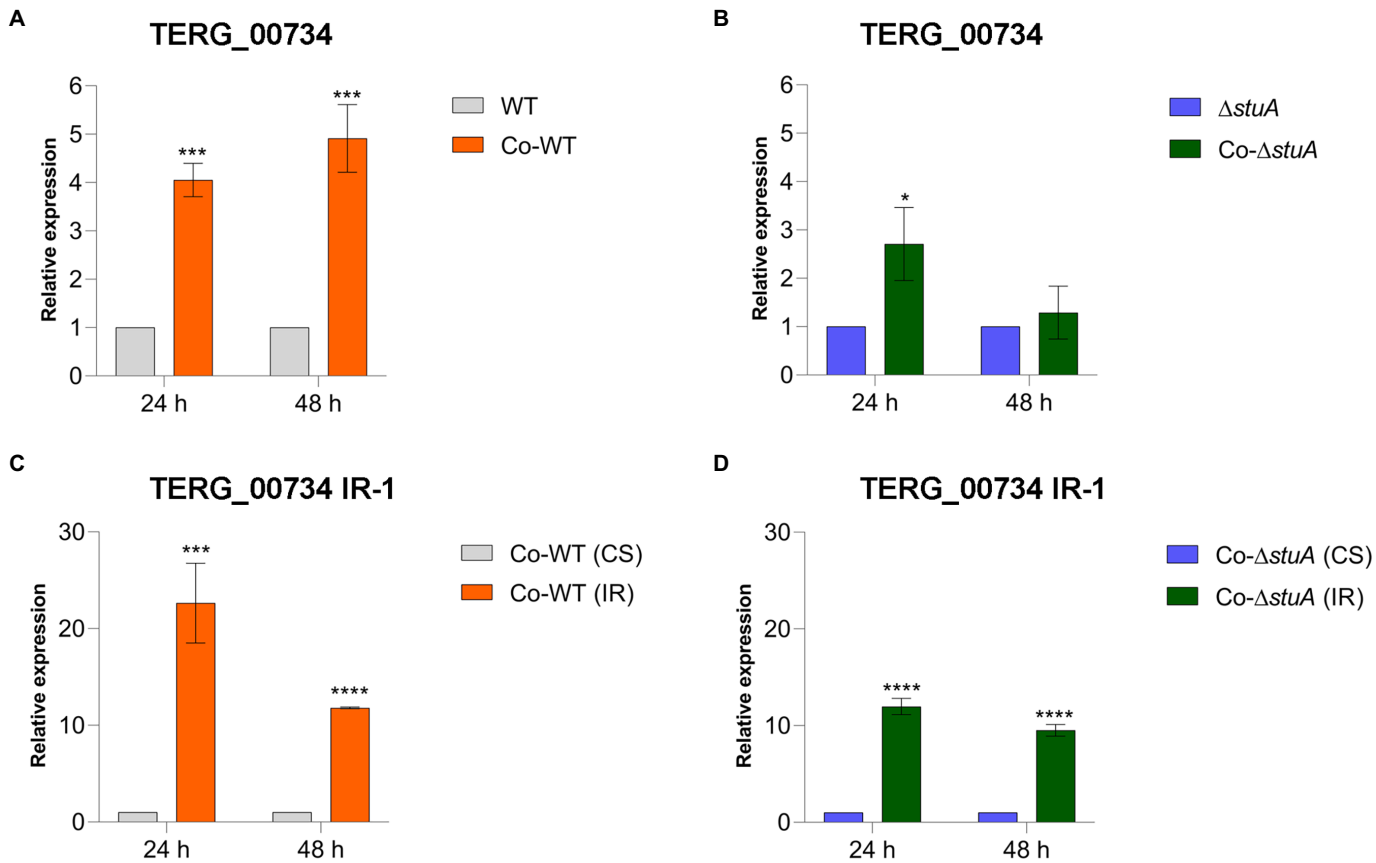
Relative expression of the CS isoform of TERG_00734 in co-culture with keratinocytes with **(A)** WT and **(B)** Δ*stuA* strain, taking the level of transcripts of the fungal cells as the reference for normalizing conditions for each strain. Relative expression of the IR isoform of TERG_00734 in co-culture with keratinocytes with **(C)** WT and **(D)** Δ*stuA* strain, taking the levels of CS transcripts for each strain as the reference of normalizing condition. Statistical analyses for transcript quantification experiments were carried out using unpaired *t*-tests, and statistical significance was determined by the Holm-Sidak method considering *p < 0.05, ***p < 0.001, and ****p < 0.0001.

To investigate the effects of StuA on peptidase expression when *T. rubrum* was co-cultured with human keratinocytes and the impact of IR events on the expression of these peptidases, we quantified the levels of transcripts of TERG_00734 and TERG_04614 after 24 and 48 h of co-culture. Regardless of the strain, the levels of the CS isoform of TERG_00734 were boosted in co-culture in comparison to the condition represented by the control (fungal cells cultured in RPMI medium without HaCaT cells), suggesting that this peptidase may be necessary in the presence of keratinocytes regardless of the role accomplished by StuA (Figures 4A,B). This increase was more prominent at 48 h of co-culture of the WT strain, whereas there were no differences in transcript levels at 48 h for the Δ*stuA* strain. As the presence of the CS isoform of this gene was first believed to be related to the success of fungal permanence in co-culture, we expected that the level of IR events detected for the same gene would not be positively modulated in the presence of keratinocytes. However, an opposite trend was observed in our results. As shown in Figures 4C,D, IR events tended to be more prevalent in the co-culture than those for CS events in the absence of StuA protein. Despite the positive modulation of IR events, they tended to diminish after 48 h of co-culture compared with the same condition for each strain after 24 h.

**Figure 5:**
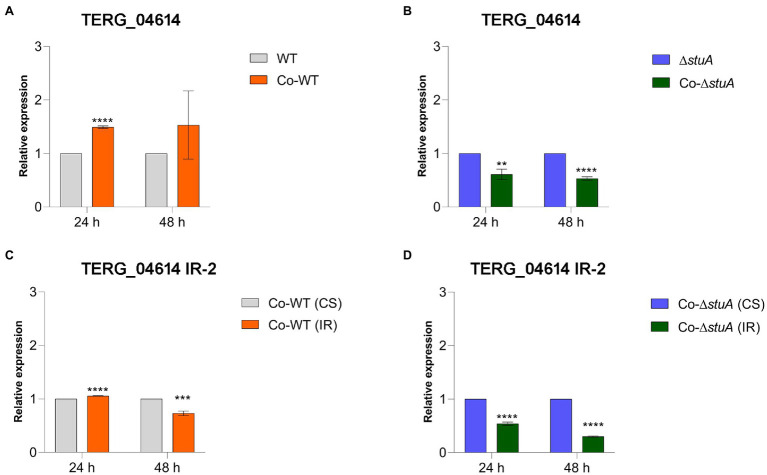
Relative expression of the CS isoform of TERG_04614 in co-culture with keratinocytes with **(A)** WT and **(B)** Δ*stuA* strain, taking the levels of the transcripts of the fungal cells as reference of normalizing condition. Relative expression of the IR isoform of TERG_04614 in co-culture with keratinocytes with **(C)** WT and **(D)** Δ*stuA* strain, taking the levels of CS transcripts for each strain as the reference of normalizing condition. Statistical analyses for transcript quantification experiments were carried out using unpaired *t*-tests, and statistical significance was determined by the Holm-Sidak method considering **p < 0.01, ***p < 0.001, and ****p < 0.0001.

CS events were also more prevalent in the co-culture of the WT strain when we quantified the level of transcripts of TERG_04614 in comparison to the control (Figure 5A). When we investigated the effect of StuA in modulating the levels of CS events for the same gene in co-culture, we found that the absence of StuA resulted in a decline in transcript levels (Figure 5B), suggesting that for this gene, despite the co-culture conditions, StuA seems to be relevant for gene activation in the face of biological requirements. IR events for TERG_04614 were observed under regular conditions in almost all the tested conditions, regardless of the strain and time of co-culture, as we were able to observe a decrease in IR transcript levels in comparison to the CS isoform transcript levels for this gene (except for the WT strain after 24 h of culture; Figures 5C,D). For TERG_04614, IR events also tended to decline after 48 h as compared with 24 h regardless of the strain.

## Discussion

Transcriptional regulation is crucial for controlling the physiological demands of the cells. Fungal cells often switch cellular responses at the expense of others responses to maintain homeostasis. This balance is pivotal for fungal viability because TFs are sensitive to changes in environmental conditions. In this sense, the regulation of molecular processes is mediated by different elements of the cellular entities. AS, as one of these entities, is a well-studied mechanism by which transcripts are processed into distinct isoforms to generate different proteins from the same pre-mRNA.

Although AS has been extensively studied, there has been no broad research regarding this mechanism in fungi. IR events, described as the retention of intron sequences that are supposed to be spliced in a pre-mRNA transcript, are likely the most prevalent AS events in fungi (Fang et al., 2020). In addition, AS mechanisms are also related to pathogenicity in fungal cells, as specific genes of human fungal pathogens can act independently on differentially expressed genes (Sieber et al., 2018).

Our work evaluated the influence of transcriptional regulation and AS mechanisms on protease gene triggering. We aimed to investigate the modulation of TF StuA by balancing the transcript levels of proteases secreted by the dermatophyte *T. rubrum*. Moreover, we addressed the occurrence of IR events in protease transcripts of this fungus, mainly when fungal cells were subjected to interaction with human keratinocytes, mimicking a host-pathogen interaction. This analysis is especially important as previous studies have demonstrated that the proteolytic activity of the Δ*stuA* mutant is significantly compromised (Lang et al., 2020; Bitencourt et al., 2021).

Our results showed that the absence of StuA could modulate the transcript levels of the peptidase IR isoform, which suggests that StuA could be related to AS event regulation. However, IR events also occurred in the presence of StuA, as was observed for the WT strain (as this strain was used as a normalizing condition for the graphs presented in the Results section). Thus, this is the first study to identify that StuA could be involved in regulating splicing machinery in *T. rubrum* and the modulation of other elements involved in IR events. Therefore, our findings support the hypotheses that the roles of AS and StuA may be independent. However, the regulation exerted by each of these entities is synergistically regulating the protease levels in *T. rubrum*.

In addition, protease expression control may be exerted at other regulatory levels, such as by small regulatory RNAs (Wang et al., 2018). These small molecules are capable of boosting or impairing translation in fungal cells and are now considered to be important mechanisms involved in the modulation of protease levels in dermatophytes (Wang et al., 2018). It is of note that the coordination accomplished by fungal cells to drive the protease balance is a multistep process that requires the involvement of several distinct levels of regulation. In the present study, our primary goal was to investigate the role of the transcription factors StuA and AS as pre-transcriptional and post-transcriptional entities, respectively, in the balance of peptidase transcript levels. However, pre-translational entities such as small regulatory RNAs were not the target of our investigation. Hence, the secretion of proteases may be due to small regulatory RNAs, as these molecules can directly influence the levels of enzymes capable of degrading keratin in host tissues.

Considering that exoproteases, such as peptidases, are necessary for keratin hydrolysis and play an essential role in nutrient assimilation for the nutrition of dermatophytes during host-pathogen interactions (Mercer and Stewart, 2019), we focused on the transcriptional regulation of one dipeptidase and one dipeptidyl peptidase of *T. rubrum*. Dipeptidyl peptidases are a group of enzymes capable of removing dipeptides from polypeptides previously generated by enzymes required for keratin degradation (Mercer and Stewart, 2019). Protease activity is crucial for dermatophyte invasion of keratinized tissues. Thus, the metabolism of keratin results in small nitrogen-rich molecules that fungi can take up as a nutritional source. A major part of the genome of dermatophytes is responsible for coding proteases and peptidases, highlighting the importance of unveiling the regulatory mechanisms that drive the transcription of such enzymes (Martinez et al., 2012; Mercer and Stewart, 2019).

As observed for transcript isoforms formed by CS or IR (Figure 1), it is also noteworthy to hypothesize that transcripts under IR events might not always result in inactive protein molecules, as could occur for TERG_04614 (Figure 1B), giving rise to different proteins which may adopt functionality and activity, when it is the case. On the contrary, for transcripts that undergo incorporation of a premature stop codon, maybe, for fungal cells, it is more suitable to transcribe IR events of a given protease transcript than to block all transcription of the CS isoform of this given gene. It is always reasonable to state that transcript balance might be subjected to more than one regulatory entity in fungal cells, as the role played by one does not abolish the role played by the other.

### StuA Modulates Proteases Gene Transcription During Growth in the Keratin-Containing Medium

By mining previous RNA sequencing results of a *T. rubrum* Δ*stuA* strain grown on keratin, we summarized the differentially expressed protease genes and presented them in Supplementary Table S3 (Bitencourt et al., 2021). It is worth mentioning that the modulation of protease transcription on keratin substrates is important for inferring the influence of proteases on fungal infections. Interestingly, the exoproteome of dermatophytes revealed that some proteases could be identified as antigenic, highlighting the relevance of proteases as virulence factors in such species (Eymann et al., 2018). Therefore, we carefully examined the influence of StuA on protease gene expression by validating transcript levels previously identified by RNA sequencing. The RT-qPCR results of our study revealed that StuA directly influenced the transcription of the peptidases TERG_00734 and TERG_04614. In contrast to findings in RNA analysis by Bitencourt et al. (2021), our results showed that the absence of StuA resulted in a decrease in the transcript levels of TERG_00734. Interestingly, it also promoted a decline in the transcript levels of keratin TERG_04614, which was not modulated after 48 h of culture. We attribute the differences in the RNA sequencing and RT-qPCR results to the sensitivity of these techniques and their analysis processing methods. This does not compromise the analysis because the influence exerted by the StuA is likely to be maintained regardless of the module intensity.

### Proteases Are Distinctively Required During Fungus–Host Interaction

As keratin is an inert substrate for *T. rubrum* interaction, we hypothesized that it would achieve a different pattern of peptidase transcription in the presence of keratinocytes. This is because they are living cells and also regulate mechanisms to maintain viability and responses against fungal attacks by signaling and recruiting immune system cells, as well as producing compounds with antimicrobial effects to avoid fungal penetration and subsequent invasion. We observed that TERG_00734 transcripts were more abundant in WT co-cultures than in the control. Moreover, the absence of StuA did not seem to cause a defective transcriptional pattern for the same gene after 24 h of co-culture, suggesting that this dipeptidase is indeed required for fungal responses in contact with keratinocytes.

However, the transcript levels of the dipeptidyl-peptidase TERG_04614 were directly affected in the absence of StuA in the co-culture. In this case, StuA is likely to be an activating TF that can potentialize the expression of the CS isoform of this protein to guarantee its role in keratin deconstruction. This effect seems to be confirmed, as higher levels of transcripts of this dipeptidyl peptidase have been detected in the co-culture of the WT strain of *T. rubrum*, particularly at 24 h of culture, a time point critical for the establishment of proteases as a way to colonize and invade host-keratinized tissues.

We hypothesized that as protease expression in Δ*stuA* co-culture was lower than in the control, it would severely impair hyphal penetration in the absence of StuA. Previous studies by our research group showed that StuA is essential for adaption, development, and cell–cell adhesion, and keratinolytic activity was altered in the absence of this TF (Lang et al., 2020; Bitencourt et al., 2021). Thus, in co-culture, lack of protease expression would elicit an inadequate response toward tissue penetration, suggesting that StuA could be a suitable target for future drug design for dermatophytosis treatment.

### AS Drives Protease Regulation in *Trichophyton rubrum* in Both Keratin-Containing Medium and Co-culture System

AS-processing events have already been described in *T. rubrum* as a relevant mechanism for regulating the expression of genes involved in important biological processes. It was described IR events occurring in determined genes as a mechanism of adaptation to avoid fatty acid toxicity in *T. rubrum* after exposure to undecanoic acid (Mendes et al., 2018). Recently, an IR event in a heat shock protein gene was described as a cell adaptation strategy for this species in response to undecanoic acid and terbinafine, an antifungal drug used to treat dermatophytosis (Neves-da-Rocha et al., 2019). In addition, a novel molecular mechanism for the activation of STE20/PakA kinase in *T. rubrum* based on an alternative pre-RNA splicing process has been suggested (Gomes et al., 2018). Moreover, deeper investigations have correlated the occurrence of AS mechanisms in human fungal pathogens, highlighting the potential of AS to participate in relevant cellular processes to maintain fungal viability (Sieber et al., 2018).

Based on these findings, we confirmed that AS modulates the transcription of proteases secreted by *T. rubrum*, as the IR isoforms of TERG_00734 and TERG_04614 were detected in our analysis (Figures 2C, 3C). The AS isoforms found in our work provide solid evidence that splicing could occur in protease transcripts as a regulatory mechanism to boost or impair the expression of such genes.

As shown in Table 1, IR events were differentially quantified after the RNA sequencing of *T. rubrum* grown on keratin (Bitencourt et al., 2021). We focused on studying only two of the most relevant proteases found to undergo AS events: TERG_00734 and TERG_04614. We hypothesized that StuA is a direct modulator of the TERG_00734 and TERG_04614 protease genes in a transcriptional level of regulation that occurs independently of AS events. Our results indicate that there must be other regulatory entities controlling such protease genes, such as AS mechanisms. Interestingly, we observed an effect of modulation accomplished by both StuA (transcriptional regulation balance) and AS (molecular regulatory balance), which interact independently. We highlight IR events in TERG_04614, whose abundance was not differentially detected by RNA sequencing after 24 or 48 h of growth in a keratin-containing medium (Supplementary Table S3). These findings suggest that, although not regulated by StuA, non-differentially expressed genes may indeed be under other regulatory mechanisms. Notably, we also found that in the co-culture with human keratinocytes, the levels of transcripts referring to the IR isoform of both proteases evaluated in our study tended to be higher in the WT cultures than in the CS isoform, mainly after 24 h (Figures 4C, 5C). However, we found that in the Δ*stuA* co-culture, IR transcript levels for proteases were higher for TERG_00734 and lower for TERG_004614 compared to the CS isoform, suggesting that the absence of StuA may not be related to a regulation pattern that is always expected to occur in the case of AS mechanisms.

The evidence found here supports the hypothesis that transcriptional and AS regulatory mechanisms do not always have opposite effects. As we observed in both keratin culture and co-culture system, the increase in CS isoform levels of a given protease might be related to the increase in IR events of the transcripts for the same protease gene, suggesting that there is a balance between the regulation of two entities aiming at fungal homeostasis. Independent AS regulation has already been reported by Sieber et al. (2018) as a relevant mechanism for fungal response to host and stress, highlighting that such a mechanism occurs to change functionality (Sieber et al., 2018).

Altogether, these findings suggest that StuA plays a precise role in coordinating protease regulation to promote keratin assimilation. It does not only act as an activator, but also as a mediator of such a balance to guarantee that only required enzymes are processed for a predetermined time, while others are not targets of cellular machinery for transcription until they are requested. Moreover, we emphasize only the direct effect of StuA on protease expression. Still, it is already known that StuA is involved in regulating genes coding for transcriptional regulation processes, which warrants a more profound elucidation of the transcriptional balance exerted by TFs over themselves (Bitencourt et al., 2021). Finally, we infer that compensation for protease gene expression in *T. rubrum* depends on both StuA and AS events to maintain fungal cell homeostasis.

## Conclusion

We described how TF, StuA, and AS regulatory mechanisms (IR events) might interact independently to promote a balance toward protease transcription. To the best of our knowledge, this is the first time that the influence of AS on peptidase regulation has been described, highlighting the relevance of studying AS as a regulatory element involved in the regulation of virulence factors in *T. rubrum*, such as protease genes. Our results suggest that the transcriptional regulation driven by StuA is directly involved in protease regulation. However, it is not the only way to modulate protease transcript levels in this dermatophyte.

## Data Availability Statement

Publicly available datasets were analyzed in this study. This data can be found at: The RNA-seq dataset used in this study is available at the Gene Expression Omnibus (http://www.ncbi.nlm.nih.gov/geo), under the accession numbers GSE163357 and GSE134406.

## Author Contributions

LM-S and MP designed, conducted all the experiments, and wrote the manuscript. PS conducted *in silico* approaches to search for splicing isoforms in RNA seq data. NM-R and AR supervised and provided scientific support and reviewed the manuscript. All authors contributed to the article and approved the submitted version.

## Funding

This work was supported by grants from the Brazilian Agencies: São Paulo Research Foundation—FAPESP (proc. no. 2019/22596-9, postdoctoral scholarships nos. 2021/10255-2 to LM-S and 2021/10359-2 to MP); National Council for Scientific and Technological Development—CNPq (grants no. 307871/2021-5 and 307876/2021-7); Coordenação de Aperfeiçoamento de Pessoal de Nível Superior (CAPES)—Finance Code 001; and Fundação de Apoio ao Ensino, Pesquisa e Assistência—FAEPA.

## Conflict of Interest

The authors declare that the research was conducted in the absence of any commercial or financial relationships that could be construed as a potential conflict of interest.

## Publisher’s Note

All claims expressed in this article are solely those of the authors and do not necessarily represent those of their affiliated organizations, or those of the publisher, the editors and the reviewers. Any product that may be evaluated in this article, or claim that may be made by its manufacturer, is not guaranteed or endorsed by the publisher.

## Footnotes

^1^ https://web.expasy.org/translate/

^2^ https://fungi.ensembl.org/

^3^ https://ensemblgenomes.org/

## Abbreviations

AS: Alternate splicing
TF: Transcription factor
IR: Intron retention
WT: Wild type
CS: Conventional splicing
