# Supplementary material for: Peptidase Regulation in Trichophyton rubrum Is Mediated by the Synergism Between Alternative Splicing and StuA-Dependent Transcriptional Mechanisms
Source: Front Microbiol. 2022 Jun 17;13:930398. doi: 10.3389/fmicb.2022.930398 (PMC9247388; doi:10.3389/fmicb.2022.930398)
Supplement: Supplementary file 1 [file Data_Sheet_1.PDF]

Supplementary Tables

Table S1. Oligonucleotides used in qPCR reactions.

Target	Primer ID	Sequence	Concentration (nM)
TERG_00734 CS	TERG_00734_P_F	TGTTCTCATTGAAGGGGAGCGGATAA	70
	TERG_00734_P_R	ATGAGTGTGAGCATCGCC	100
TERG_00734 IR	TERG_00734 IR-1_F	GTTCTCATTGAAGGTATGTCATGTTTAGCC	300
	TERG_00734 IR-1_R	TCCCCTGTACCAATCGTTAATC	300
TERG_04614 CS	TERG_04614_P_F	GCAATGGTCAACCTGGAG	100
	TERG_04614_P_R	TCTCAAATTCCTTGCCCCAG	100
TERG_04614 IR	TERG_04614 IR-2_F	AGCAATGGTAACGTATCTACCC	200
	TERG_04614 IR-2_R	CTTCTCAGCATTCTCCACCAG	200
gapdh	gapdh_F	GCGTGACCCAGCCAACA	200
	gapdh_R	CGGTGGACTTCGACGATGTAGT	200

CS – Conventional splicing isoform. IR – Intron retention event.
F and R represent forward and reverse orientation, respectively.

Table S2. Oligonucleotides used in conventional PCR reactions.

	AS event	Primers	Sequence	Concentration (nM)	Target DNA	Amplicon (pb)
TERG_00734	CS	TERG_00734_P_F	TGTTCTCATTGAAGGGGAGCGGATAA	200	processed mRNA	139
		TERG_00734_P_R	ATGAGTGTGAGCATCGCC	200		
	IR	TERG_00734 IR-1_F	ACCCTACCGTGGTGATG	200	processed mRNA	111
		TERG_00734 IR-1_R	CGTCCAAGGATCTTCCGTAC	200		
		TERG_00734 IR-1_F	ACCCTACCGTGGTGATG	200	intronic transcripts	194
		TERG_00734 IR-1_R	CGTCCAAGGATCTTCCGTAC	200		
TERG_04614	CS	TERG_04614_P_F	GCAATGGTCAACCTGGAG	200	processed mRNA	97
		TERG_04614_P_R	TCTCAAATTCCTTGCCCCAG	200		
	IR	TERG_04614 IR-2_F	TTGGCTTTGTCGAGACCTA	200	processed mRNA	136
		TERG_04614 IR-2_R	AGCTTGGGGATCATCTTCTC	200		
		TERG_04614 IR-2_F	TTGGCTTTGTCGAGACCTA	200	intronic transcripts	208
		TERG_04614 IR-2_R	AGCTTGGGGATCATCTTCTC	200		

CS – Conventional splicing isoform. IR – Intron retention event. F and R represent forward and reverse orientation, respectively.

Table S3. Gene expression response of proteases modulated in Δ*stuA* grown in keratin at 24, 48 and 96 h, shown by previous RNA-sequencing*

**ID**	**24 hours**	**48 hours**	**96 hours**	**Gene Product Name**
TERG_06767	2.63	2.52	1.62	Aminopeptidase (*T. tonsurans*)
TERG_00734	1.99	2.07	-	Dipeptidase (*T. tonsurans*)
TERG_08405	-	-	1.95	Leucine aminopeptidase 2
TERG_03248	1.19	1.19	-	Extracelular metalloproteinase 3
TERG_05735	-	1.06	1.43	Dipeptidyl peptidase 4
TERG_08201	-	1.09	-	Subtilisin-like protease 5
TERG_04411	1.04	0.61	-	Dipeptidase (*T. equinum*)
TERG_00380	-	0.86	-	Peptidase M28 family protein (*T. equinum*)
TERG_03815	-	0.82	-	Subtilisin- like protease 3
TERG_07085	-	-0.57	-	Peptidase (*T. tonsurans*)
TERG_08557	-	-0.94	-1.86	Carboxypeptidase S1, putative (*A. benhamiae*)
TERG_12591	-	-1.01	-	Peptidase S8 family protein (*T. equinum*)
TERG_11593	-1.13	-1.77	-1.51	Carboxypeptidase Y, putative (*A. benhamiae*)
TERG_04614	-	-	-1.24	Dipeptidyl peptidase 3
TERG_11594	-	-1.27	-1.41	Serine Carboxypeptidase
TERG_01134	-1.28	-1.28	-	Metalloprotease MEP1 (*A. benhamiae*)
TERG_01840	-2.72	-2.27	-2.15	Carboxypeptidase S1 (*T. equinum)*
TERG_05652	-3.31	-3.15	-	Leucine aminopeptidase 1

* The generated RNA-seq dataset is available at the Gene Expression Omnibus (http://www.ncbi.nlm.nih.gov/geo), under the accession numbers GSE163357 and GSE134406.

Gene expression values are expressed in Log_2 Fold Change
